# Unpredicted clinical manifestation of COVID-19: a unique case report and review of literature

**DOI:** 10.1186/s43163-022-00312-z

**Published:** 2022-09-07

**Authors:** Raid M. Al-Ani, Rasheed Ali Rashid

**Affiliations:** 1 Department of Surgery/Otolaryngology, College of Medicine, University Of Anbar, Ramadi, Iraq; 2 Department of Surgery/Otolaryngology, College of Medicine, Tikrit University, Tikrit, Iraq

**Keywords:** COVID-19, Supraclavicular lymph node, Cervical lymphadenopathy, Case report

## Abstract

**Background:**

Cervical lymphadenopathy in children is a common problem in daily clinical practice. Many cases of cervical lymphadenopathy after the COVID-19 vaccine were reported. However, there is no yet reporting a case of supraclavicular cervical lymphadenopathy due to COVID-19.

**Case presentation:**

A 12-year-old girl presented with fever, cough, fatigue, anosmia, and ageusia. COVID-19 was confirmed by real-time PCR. The symptoms were resolved within 10 days. Seven days later, she complained of supraclavicular swelling. Physical examination revealed painless, multiple, and mobile supraclavicular lymph nodes. Ultrasound and fine-needle aspiration cytology were suspicious. Therefore, an excisional biopsy of the largest node was performed. The specimen was sent for histopathology and immunohistochemistry evaluation which confirmed the benign nature of the lymph node.

**Conclusion:**

To our best knowledge, this is the first case of supraclavicular lymphadenopathy in a child with COVID-19. It is essential to put COVID-19 in the differential diagnosis of cervical lymphadenopathy.

## Background

There are various otorhinolaryngological manifestations as a result of the COVID-19 pandemic, including, but not limited to, smell and taste abnormalities, dysphonia, hearing loss, sore throat, nasal obstruction, and parotitis [1–3].

The neck is the joining part between the head and body. There are various causes of neck masses; these are broadly divided into three groups congenital or developmental, inflammatory (infectious or noninfectious), and tumors [benign or malignant (primary or secondary)].

Many viruses, particularly in the pediatric population, may cause cervical lymphadenopathies like adenovirus, Epstein-Barr virus, herpes virus, coxsackievirus, and cytomegalovirus. Moreover, cervical lymphadenopathy following COVID-19 vaccines was reported [4, 5]. Distinguin et al. reported three COVID-19 patients with cervical lymphadenopathy in level 2 (upper jugular group) [6]. However, there is no reported case of supraclavicular cervical lymphadenopathy due to COVID-19. We reported the first case of supraclavicular lymph node enlargement in a 12-year-old girl following COVID-19.

## Case presentation

A 12-year-old female presented with a neck mass in the right supraclavicular area of 1-month duration. The patient had a history of contact with her infected grandmother with COVID-19. She complained of fever (38.5 °C), dry cough, fatigue, and loss of smell and taste. She consulted a doctor and gave her advice on bed rest and home quarantine from her family members after sending her for a real-time polymerase chain reaction (PCR) test of the nasopharyngeal swab. The test was positive for severe acute respiratory syndrome coronavirus 2 (SARS-CoV-2). Chest X-ray revealed a right-sided upper zone pneumonic patch. Supportive treatment in the form of antipyretic and tonic as well as antibiotics (azithromycin) was given as per the protocol of COVID-19 treatment approved by the Iraqi Ministry of Health. The patient became well, and all presenting symptoms disappeared entirely during the 10-day follow-up.

**Fig. 1:**
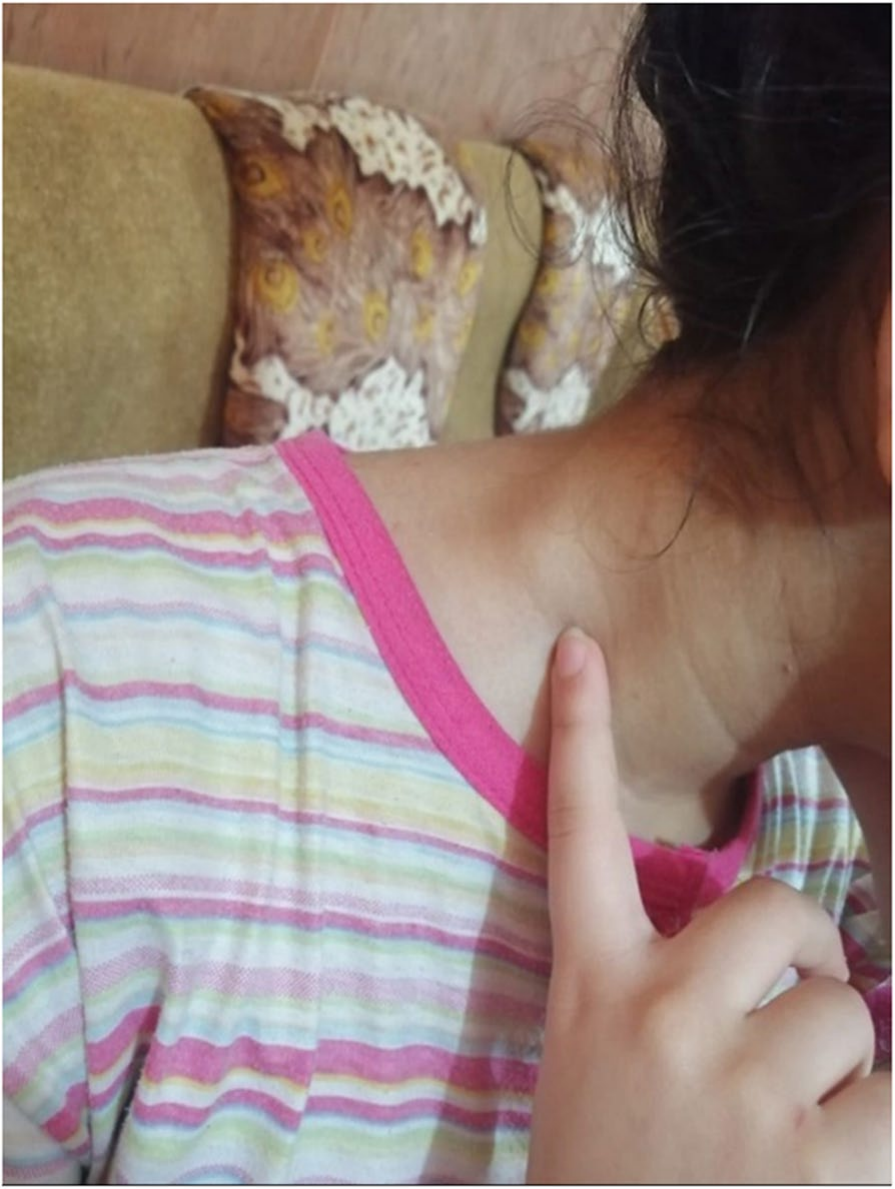
Showing the right side of the neck with supraclavicular swelling in a 12-year-old girl

**Fig. 2:**
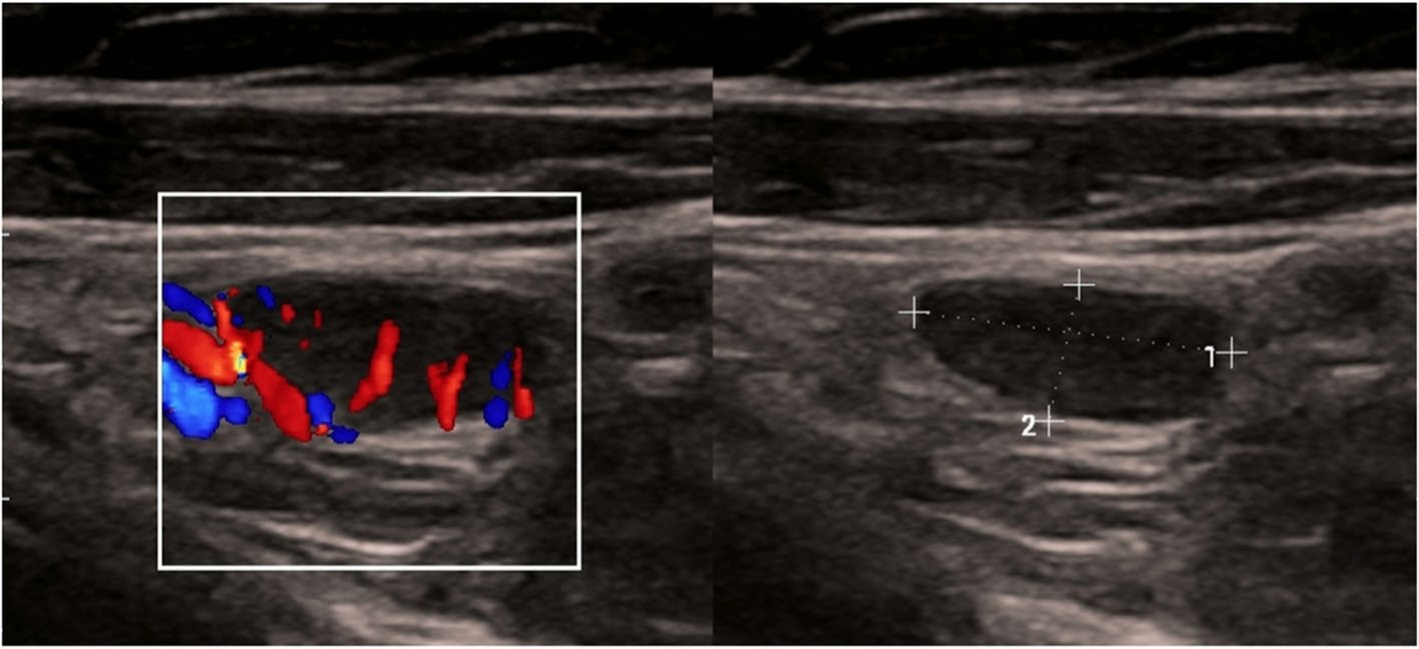
Grayscale ultrasound of the neck shows abnormal-looking lymph nodes evident by exaggerated hypoechoic echotexture and lost fatty hilum. The largest one 18 × 10 mm in diameter

**Fig. 3:**
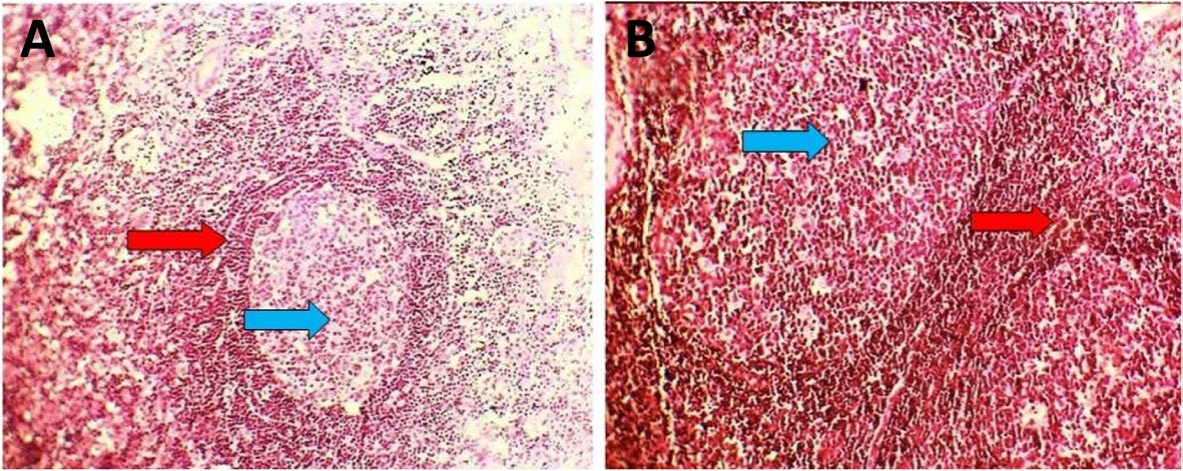
Showing a benign reactive lymph node, which has a mantle zone (red arrow) that is surrounding a pale germinal center (blue arrow). **A** H&E × 40 and **B** H&E × 100

**Fig. 4:**
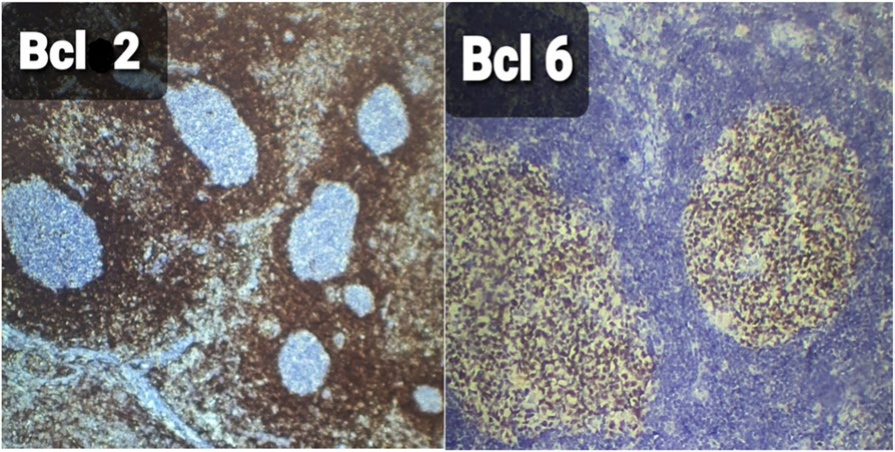
Showing positive immunohistochemical (IHC) expression of BCL-2 in the mantle zones and negative brownish discoloration of the nuclear and cytoplasmic stain. Besides, positive IHC expression of BCL-6 in the germinal centers of the lymph node with reactive hyperplasia, with a brownish discoloration of the nuclear stain

One week later, a right supraclavicular lump appeared; it was painless and gradually increased in size (Fig. 1). Physical examination revealed a non-tender neck mass in the right supraclavicular region, oval in shape, 2 × 1.5 cm, freely mobile, with no scar, and no skin changes over the swelling or surrounding areas. There were no focal infective areas, masses, or other lymph node enlargement in the body. The patient took a 5-day antibiotic course but without benefit. Ultrasound examination revealed multiple cervical lymph nodes in the right supraclavicular area, the largest of 18 × 10 mm in diameter. These nodes showed abnormal fatty hilum, abnormal round index, and exaggerated hypo-echoic texture, as shown in Fig. 2. The possible differential diagnosis could be infectious mononucleosis, toxoplasmosis, cytomegalovirus infection, and less likely tuberculosis or lymphoma. No abnormalities were found on abdominal and axillary sonographic examination. Laboratory tests revealed all are normal apart from high IgM against SARS-CoV-2 and lymphocytosis. Fine needle aspiration cytology revealed a suspicion of abnormal cells. Excisional biopsy was subjected to histopathological examination and immunohistochemistry study. These examinations revealed reactive hyperplasia with no abnormal cells (Figs. 3 and 4). There was no lesion recurrence at the 2-month follow-up visit, and the patient made her normal daily living well. The possible cause of her neck swelling was COVID-19 owing to the patient’s clinical presentation, positive real-time PCR test of the nasopharyngeal swab, the result of the serological test of SARS-CoV-2, and no features on physical examination, and investigation supported other causes as listed in the differential diagnosis. The parents gave informed consent to publish the case.

## Discussion

The head and neck contain around 2/3rd of the lymph nodes in the body. Besides, the inflammatory or malignant process in any area can reach the neck through the lymphatic system. Therefore, there is a huge list of causes of cervical lymphadenopathy (enlargement of a node > 1 cm in diameter) [7]. Cervical lymphadenopathy is common in the pediatric population, and most of the cases are benign. The first systematic review about the causes of cervical lymphadenopathy in children by Deosthali et al. [7] reported that 67.8% of the 2687 cases are due to nonspecific benign causes, followed by Epstein-Barr virus (8.86%), malignancy (4.69%), and granulomatous disease (4.06%). In the presenting case, the histopathology and immunohistochemistry evaluations revealed the reactive benign nature of the supraclavicular lymph node. The high possible cause of this cervical lymphadenopathy was COVID-19 because the patient was diagnosed as COVID-19 by real-time PCR of the nasopharyngeal swab and high IgM as well as an absence of indicators of other pathologies in the history, examination, and investigations. Accordingly, COVID-19 can lead to reactive cervical lymph node enlargement.

Involvement of the axillary and/or supraclavicular lymph nodes on the same side is a frequent adverse effect of the vaccines against COVID-19. This is due to local activation of the immune response [8–10]. Moreover, Distinguin et al. reported 3 cases of cervical lymphadenopathy in group 2 (upper jugular group) on magnetic resonance imaging (MRI) in patients with COVID-19. All those patients have complained of otorhinological symptoms (anosmia, aguesia, nasal obstruction, rhinorrhea, and sore throat). These symptoms are due to inflammation of the nose, nasopharynx, and oropharynx caused by SARS-CoV-2. As a result of this inflammation, a local immune reaction occurs, resulting in lymph node enlargement of the Waldeyer’s ring, neck, and parotid regions [6]. Interestingly, we presented the first case in the world of unilateral supraclavicular enlargement in a patient with COVID-19. Although the mechanism of supraclavicular lymphadenopathy is not yet known, it is necessary to put COVID-19 in the differential diagnosis of supraclavicular lymphadenopathy.

Identifying the possible ways of transmitting the SARS-CoV-2 has a major role in understanding the mechanism of the infection with its further treatment options. The specific coronavirus receptor (ACE-2 receptor) is distributed in all body tissues, including the lymph nodes [11]; therefore, it is possible to find the virus in the lymph node as in the presenting case, leading to inflammation and enlargement of the node. Another possible mechanism of getting enlargement of the supraclavicular lymph node is a local immune response in the lung.

According to the American College of Radiology (ACR) recommendations, chest X-rays and computerized tomography (CT) should not be used as a screening or first diagnostic tool for COVID-19 owing to the similarity of the radiological signs among various lung conditions. However, radiological investigation in the pediatric population plays an essential tool for moderate and severe forms of COVID-19 (as a baseline, assessment of complications, and assessment of treatment response or progression of the disease). Moreover, a chest X-ray is considered the first radiological investigation in children, and a CT scan is reserved for suspicious cases of pulmonary embolism or worsening clinical conditions [12]. Pulmonary abnormalities in children are unilateral in 55% and bilateral in 45% of affected children [13], and about 8% are affected in the right upper lobe of the lung [14], a similar finding in our patient.

Cervical lymphadenopathy following COVID-19 is uncommon. However, it can be put on the long list of differential diagnoses, including infections, primary tumor (lymphoma), or secondary malignant lymph node. Radiological investigations in the form of sonography or MRI as a diagnostic tool should be performed when there are suspicious findings on physical examination.

As reported in the literature, supraclavicular lymphadenopathy after taking the COVID-19 vaccine affects mostly females and occurs in up to 24 days (mostly in the first 10–15 days). It gradually subsides within 4–6 weeks [15]. Our case presented with features of COVID-19 (fever, fatigue, cough, and loss of smell and taste) for 10 days, and the disease was confirmed by a real-time PCR test of the nasopharyngeal swab. Seven days following the resolution of the symptoms, right supraclavicular lymphadenopathy appeared. Supraclavicular lymphadenopathy in children, particularly if persists for more than 2 weeks, carries a sinister pathology [16]. Therefore, we subjected the child to an excisional biopsy which revealed a benign nature of the lesion on the histopathological and immunohistochemical evaluation. Accordingly, the supraclavicular lymphadenopathy in the presenting case was highly suspicious that COVID-19 was a cause.

## Conclusion

Supraclavicular lymphadenopathy due to COVID-19 was not reported in the literature. However, it might be a side effect of the COVID-19 vaccine. Besides, cervical lymphadenopathy in level 2 was reported. This is the first case in the world of supraclavicular lymphadenopathy due to COVID-19 in a child. Therefore, it is necessary to ask the patients with cervical lymphadenopathy whether they have gotten COVID-19 recently.

## Data Availability

Data sharing is not applicable to this article as no datasets were generated or analysed during the current study.
